# The impact of acute thermal stress on the metabolome of the black rockfish (*Sebastes schlegelii*)

**DOI:** 10.1371/journal.pone.0217133

**Published:** 2019-05-24

**Authors:** Min Song, Ji Zhao, Hai-Shen Wen, Yun Li, Ji-Fang Li, Lan-Min Li, Ya-Xiong Tao

**Affiliations:** 1 Key Laboratory of Mariculture (Ocean University of China), Ministry of Education, Ocean University of China, Qingdao, P. R. China; 2 Department of Anatomy, Physiology and Pharmacology, College of Veterinary Medicine, Auburn University, Auburn, AL, United States of America; Universidade de Vigo, SPAIN

## Abstract

Acute change in water temperature causes heavy economic losses in the aquaculture industry. The present study investigated the metabolic and molecular effects of acute thermal stress on black rockfish (*Sebastes schlegelii*). Gas chromatography time-of-flight mass spectrometry (GC-TOF-MS)-based metabolomics was used to investigate the global metabolic response of black rockfish at a high water temperature (27°C), low water temperature (5°C) and normal water temperature (16°C). Metabolites involved in energy metabolism and basic amino acids were significantly increased upon acute exposure to 27°C (*P* < 0.05), and no change in metabolite levels occurred in the low water temperature group. However, certain fatty acid levels were elevated after cold stress (P < 0.05), and this effect was not observed in the 27°C group, suggesting that acute high and low temperature exposures caused different physiological responses. Using quantitative real-time PCR, we analyzed the expression of *ubiquitin* (*ub*), *hypoxia-inducible factor* (*hif*), *lactate dehydrogenase* (*ldh*), and *acetyl-CoA carboxylase* (*acac*). Higher expression levels of *ub*, *hif*, and *ldh* (P < 0.05) were observed in the high water temperature group, but no changes in these expression levels occurred in the low water temperature group. Our findings provide a potential metabolic profile for black rockfish when exposed to acute temperature stress and provide some insights into host metabolic and molecular responses to thermal stress.

## Introduction

With global warming, extreme climate events are predicted to increase in frequency and magnitude, intensifying the loss of fish production, impacting fishery- and aquaculture-based livelihoods, and damaging marine ecosystems [1–3]. An important consequence of extreme climatic events such as heat waves and cold currents is the quick change of water temperature. Because of the intolerance of some commercial fishes to acute thermal stress, these extreme climatic events cause severe losses for the aquaculture industry along the coast of China. Temperature change often occurs in the offshore waters of Qingdao. Black rockfish can survive temperatures ranging from 5 °C to 28 °C, with the optimal temperature ranging from 18 °C to 24 °C [4]. To reduce the economic and ecological losses caused by sudden thermal stress, a better understanding of the behavioral, physiological and genetic responses of fish to thermal stress is essential. Several physiological indictors such as blood plasma cortisol, glucose, and alanine, have been identified [5, 6]. As in other vertebrates [7], heat-shock proteins (HSPs) play important roles in overcoming adverse high thermal stress in fish [8, 9]. Through advanced transcriptional technology, multiple genes related to low thermal acclimation of fish have been identified [10, 11]. However, changes in the metabolic profiles of fish caused by environmental stress have not received sufficient attention.

Metabolomics is the study of the low molecular weight metabolites (typically < 1000 Da) within a cell, tissue or biofluid [12]. The profile of metabolites within a biological sample can provide considerable insights into the response of an organism to disease, toxicity, environmental stimulus or genetic manipulation [13, 14]. Fish metabolomics have been studied in several species for different areas of research [15]. NMR-based metabolomics, due to comparatively low resolution, is useful for examining fewer metabolites. GS-MS-based or LC-MS-based detection technologies can successfully compensate for this deficiency. Zhao et al. found that L-proline increased survival of tilapia infected by *Streptococcus agalactiae* in higher water temperature through GS-MS-based metabolomics [16]. Using LC-MS, Yan and colleagues studied changes in serum polar lipids in two common cage-cultured fishes, yellow croaker (*Pseudosciaena crocea*) and Japanese seabass (*Lateolabrax japonicas*), after a tropical storm [17]. The experiment (Ege & Krogh 1914) produced a roughly exponential decrease in respiration rate as the temperature was lowered, and this relationship became known as Krogh's normal curve [18]. When fish are confronted with seasonal cooling, they always slow their physiological processes by submitting to the Q10 effects [19], depress metabolic function by enhancing thermal effects (hibernation, torpor) or offset the Q10 effects by maintaining capacities using perfect or partial compensation [20, 21]. Ubiquitin is a small regulatory protein discovered in 1975 that plays a key role in protein degradation [22]. HIFs play essential roles in oxygen homeostasis [23]. Lactate dehydrogenases (LDHs) play important roles in several metabolic pathways, which form the center of a delicately balanced equilibrium between catabolism and anabolism of carbohydrates [24]. These enzymes catalyze the reciprocal conversion between pyruvate and lactate. ACAC is a key enzyme for lipid metabolism, responsible for converting acetyl-CoA to malonyl-CoA, which in turn is used to add to the growing acyl chain [25].We present herein the first metabolomics study on the response to thermal stress in black rockfish.

As a viviparous fish inhabiting in shallow costal water and distributed around Korea, Japan and northern China [26], black rockfish have gradually become an important species in marine cage aquaculture. Male and female black rockfish gonadal maturation occurs at different times of the year, September—November and May—June, respectively [27]. In these two periods, seawater temperature changes dramatically and extreme weather frequently occurs in the offshore areas of the Yellow Sea of China, causing black rockfish to suffer from rapid thermal change. In this study, we attempt to clarify the overall physiological response of black rockfish to acute thermal stress by employing the holistic basic analysis method of GC-TOF-MS-based metabolomics.

## Materials and methods

### Fish and temporal rearing conditions

Fifty mature male black rockfish (body length: 28.6 ± 1.4 cm, body weight: 1324.4 ± 120.7 g) were purchased from Nanshan Fisheries Market (Qingdao, China), originating from cages located in the northern Yellow Sea. Fish were kept at a density of 10 individuals in round open-circuit plastic tanks (diameter of 1 m and height 1.5 m) with aeration. The salinity of seawater was 30 ppm and the seawater had been processed through a series of purifications. The fish were held under controlled conditions (16°C water temperature and 12:12 h light: dark photoperiod). The black rockfish were anesthetized in a 0.01% solution of tricaine methane sulfonate (MS-222) before decapitation and sampling. All animal experiments were conducted in accordance with the guidelines and approval of the Animal Research and Ethics Committees of Ocean University of China. Water quality was monitored regularly. Before the thermal stress experiment, the fish were reared in the above environment for seven days.

The usage of fish was in strict accordance with the recommendations of the Guidelines for the Use of Experimental Animals of Ocean University of China. The protocol for animal care and handling used in this study was approved by the Committee on the Ethics of Animal Experiments of Ocean University of China (Permit Number: 20141201). Before sacrificing and handling, experimental fish were anesthetized with 100 ng/ml of ethyl 3-aminobenzoate methanesulfonic acid (MS222, Sigma, USA), and all efforts were made to minimize suffering. The field studies did not involve endangered or protected species.

### Acute thermal stress procedures

A total of 30 male black rockfish were used in this experiment. Based on preliminary experiments, three groups were used: a control group (16°C), low temperature group (5°C) and high temperature group (27°C). Sea water ice was used to decrease the water temperature and an electric heating rod (1000 watts) was used to increase the water temperature. After water temperature was stabilized, fish were transferred into the stress tank directly and stressed for 12 h. Blood was collected from the caudal vein by a 1-mL syringe and centrifuged (12000 r/min) after storage for 12 h at 4°C. Liver, brain, pituitary, gonad, kidney and muscle tissues were collected from each fish and immediately snap-frozen in liquid nitrogen and stored at -80°C until further analysis. A week later, samples were analyzed.

### GC-TOF-MS measurements

Six serum samples from each group were analyzed with GC-TOS-MS-based equipment. The analysis was carried out in two stages—sample preparation and detection. A serum sample of 100 μL was added into 1.5 mL Eppendorf tubes and 0.35 mL methanol and 20 μL of L-2-chlorophenylalanine (Hengbai Biotech Co, Shanghai, China) was used as an internal standard, and the sample was then stirred for 30 s and centrifuged for 15 min at 12000 rpm at 4°C. Then, 0.4 mL supernatant was transferred into a fresh 2 mL GC/MS glass vial and 21 μL of supernatant from each sample was pooled as a quality control sample, followed by drying in a vacuum concentrator without heating, and derivatization of acidic protons through an initial incubation of 20 min at 80°C with 60 μL methoxyamination reagent (20 mg/mL in pyridine) and a subsequent incubation for 2 h at 70°C with 80 μL of BSTFA reagent (Regis Technologies, Morton Grove, IL, USA). Samples were then cooled to room temperature and mixed well for GC-TOF-MS analysis.

GC-TOF-MS analysis was performed using an Agilent 7890 gas chromatograph system (Agilent, Santa Clara, CA, USA) coupled with a Pegasus HT time-of-flight mass spectrometer (LECO, St. Joseph, MI, USA). The system utilized a DB-5 MS capillary column coated with 5% diphenyl cross-linked with 95% dimethylpolysiloxane (30 m × 250μm inner diameter, 0.25 μm film thickness; J&W Scientific, Folsom, CA, USA). A 1 μL aliquot of the analyte was injected in splitless mode. Helium was used as the carrier gas, the front inlet purge flow was 3 mL min^−1^, and the gas flow rate through the column was 20 mL min^−1^. The initial temperature was kept at 50°C for 1 min, then raised to 320°C at a rate of 20°C min^−1^, and kept at 320°C for 5 min. The injection, transfer line, and ion source temperatures were 280, 280, and 250°C, respectively. The energy was -70 eV in electron impact mode. The mass spectrometry data were acquired in full-scan mode with the m/z range of 85–600 at a rate of 20 spectra per second after a solvent delay of 4.93 min.

### Quantitative RT-PCR gene expression measurements

To explore genes that control the metabolic responses to thermal stress, expression levels of several genes, including *ubiquitin* (*ub*), *hypoxia-inducible factor (hif) 1α* (*hif1α)*, *hif2α*, *lactate dehydrogenase* (*ldh*), *acetyl-CoA carboxylase* (*acac*) *1* (*acac1*), and *acac2* were determined by quantitative RT-PCR (qRT-PCR). Gene-specific primers (**S1 Table**) were designed using the Primer6 software (PREMIER Biosoft International, Palo Alto CA, USA).

Total RNA was isolated using TRIzol (Invitrogen, California, Carlsbad, USA) reagent and treated with PrimeScript Real Time reagent kit with genomic DNA Eraser (Takara, Kusatsu, Shiga, Japan) to remove genomic DNA and synthesize cDNA. The concentration and integrity of total RNA were assessed by Nanodrop ND-1000 spectrophotometry and gel electrophoresis (1% agarose, 0.6 l g/mL ethidium bromide). The cDNA samples were analyzed with an ABI StepOne Plus Real-Time PCR system (Applied Biosystems) using SYBR Premix Ex Taq (Takara), 2 μl of template cDNA, 0.4 μl of each primer, 10 μl of SYBR I Master Mix, 0.4 μl of ROX and 6.8 μl of nuclease-free water in a total reaction volume of 20 μl. All cDNA products were diluted to 500 ng/μl. The qRT-PCR cycling parameters were 95°C for 30 s followed by 40 cycles of 95°C for 5 s and the primer annealing temperature for 30 s followed by a melting curve analysis from 65°C to 95°C according to the protocol of the reagent kit. A control without template DNA was included on every plate to rule out nonspecific contamination, for example, genomic DNA contamination, while the dissociation curve analysis was used to verify primer specificity. A standard dilution curve was set up for each primer pair to obtain the best amplification efficiency and the suitable dilution concentration for subsequent experiments. *18S* ribosomal RNA (*18S*) was used as the reference gene to normalize the expression analysis and the 2^-ΔΔCT^ method was used to analyze the expression levels of target genes [28].

### Data preprocessing and statistical analyses

Chroma TOF4.3X software of the LECO Corporation and the LECO-Fiehn Rtx5 database were used for raw peak extraction, data baselines filtration and calibration of the baseline, peak alignment, deconvolution analysis, peak identification and integration of the peak area. The RI (retention time index) method was used for peak identification, and the RI tolerance was 5000. Then, the raw peaks were left through the interquartile range denoising method, and missing values of raw data were filled up by half of the minimum value.

The resulting three-dimensional data, including the peak number, sample name, and normalized peak area, were processed with the SIMCA14 software package (Umetrics, Umea, Sweden) for principal component analysis (PCA) and orthogonal projections to latent structures-discriminant analysis (OPLS-DA). Seven-fold cross validation was used to estimate the robustness and the predictive ability of the model. The selection of changed metabolites was defined by the limiting condition that the first principal component of the variable importance projection (VIP) of metabolites exceeded 1.0 with Student's T test (P > 0.05). In addition, commercial databases, including KEGG (http://www.genome.jp/kegg/) and NIST (http://www.nist.gov/index.html), were utilized to search for the pathways of the metabolites. Heml 1.0 and R language were used to make heat map and pathways enrichment figures.

Gene expression and metabolite data were determined by SPSS 13.0, one-way ANOVA followed by Duncan’s multiple range tests, and differences were accepted as statistically significance at P < 0.05.

## Results

### Metabolic profile of black rockfish under different acute water temperature changes

**Fig 1:**
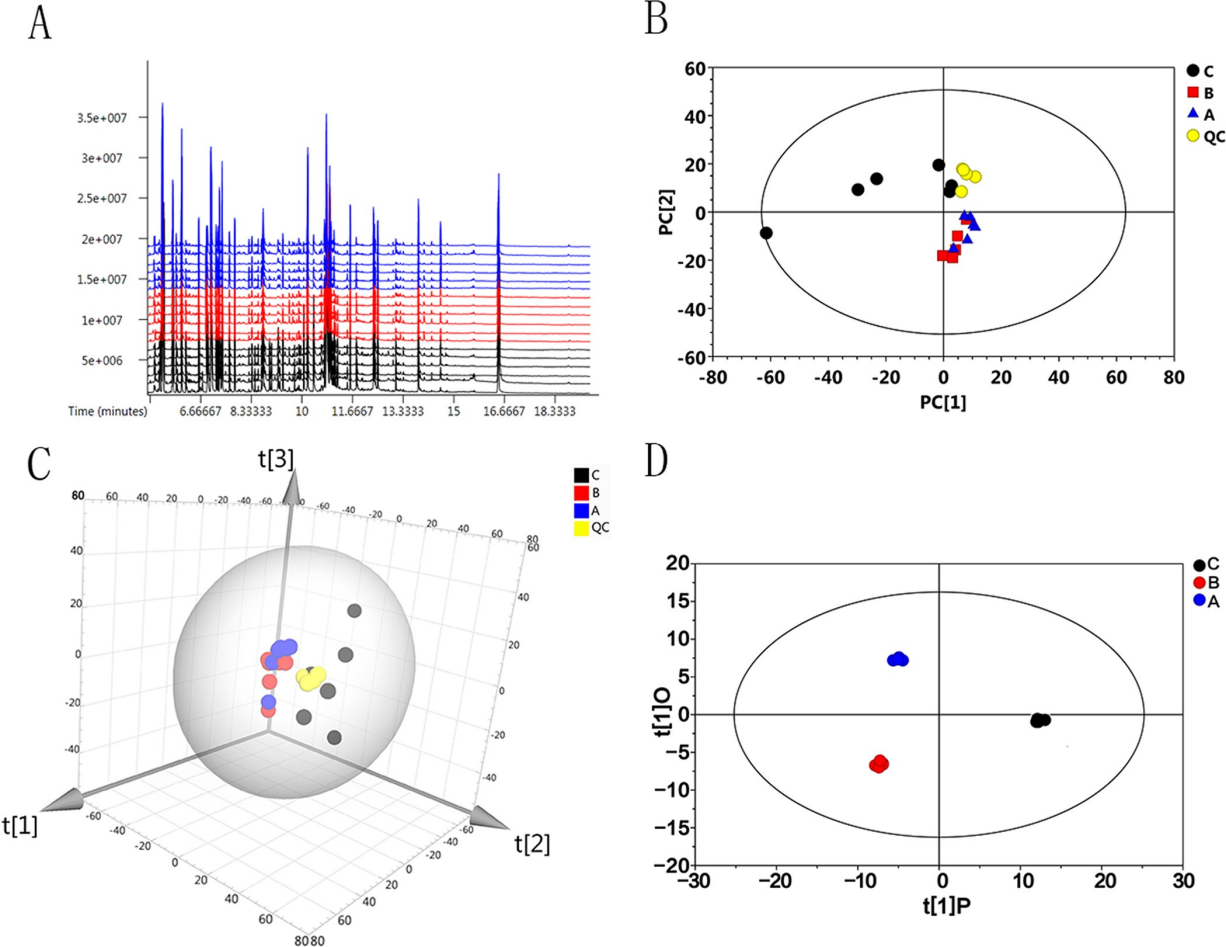
Serum metabolic profile of black rockfish exposed to different water temperatures obtained through GC-MS-TOF-based metabolomics and multivariate data analysis. A, total ion current chromatograms (TIC) of all samples. B, the two-dimensional scatter plot of the PCA model of three thermal groups and QC samples. C, the three-dimensional scatter plot of the PCA model of three thermal groups and QC samples. D, the scatter plot of the OPLS-DA model. The A, B and C labels in figure represent control group (16°C), low temperature group (5°C) and high temperature group (27°C), respectively.

Typical total ion current chromatograms (TIC) of all samples are shown in **Fig 1A**. The uniformity of the retention time in each group indicated the stability of the instrument and the reliability of the data. A total of 614 peaks were obtained from raw data and 567 peaks were obtained after denoising. The LECO/Fiehn Metabolomics Library allowed us to identify 260 metabolites. To differentiate between the different groups, the data were first subjected to PCA with the SIMCA14 software package. The two-dimensional and three-dimensional scatter plots of the PCA model of the three groups are shown in **Fig 1B** and **1C**. The five QC samples that are tightly localized in the figure also implied the compatibility of the PCA model. The discrete distribution of the 27°C group was likely due to high biological variation. Visual inspection of the PCA model also revealed no significant trend between the 27 and 5°C groups, suggesting the presence of nonrelated variables because PCA is an unbiased multivariate data analysis.

Furthermore, supervised OPLS-DA was applied and shown in **Fig 1D**. This model filters the orthogonal signals with regards to the Y variable (the thermal variable) and leaves the highly corrected signals to the Y variable [29], therefore we were able to obtain a superior model. The OPLS-DA model perfectly discriminated the three groups, revealing significant changes in the concentrations of metabolites in the serum of black rockfish when the water temperature changed sharply.

### Differential metabolites in response to high and low water temperature

**Fig 2:**
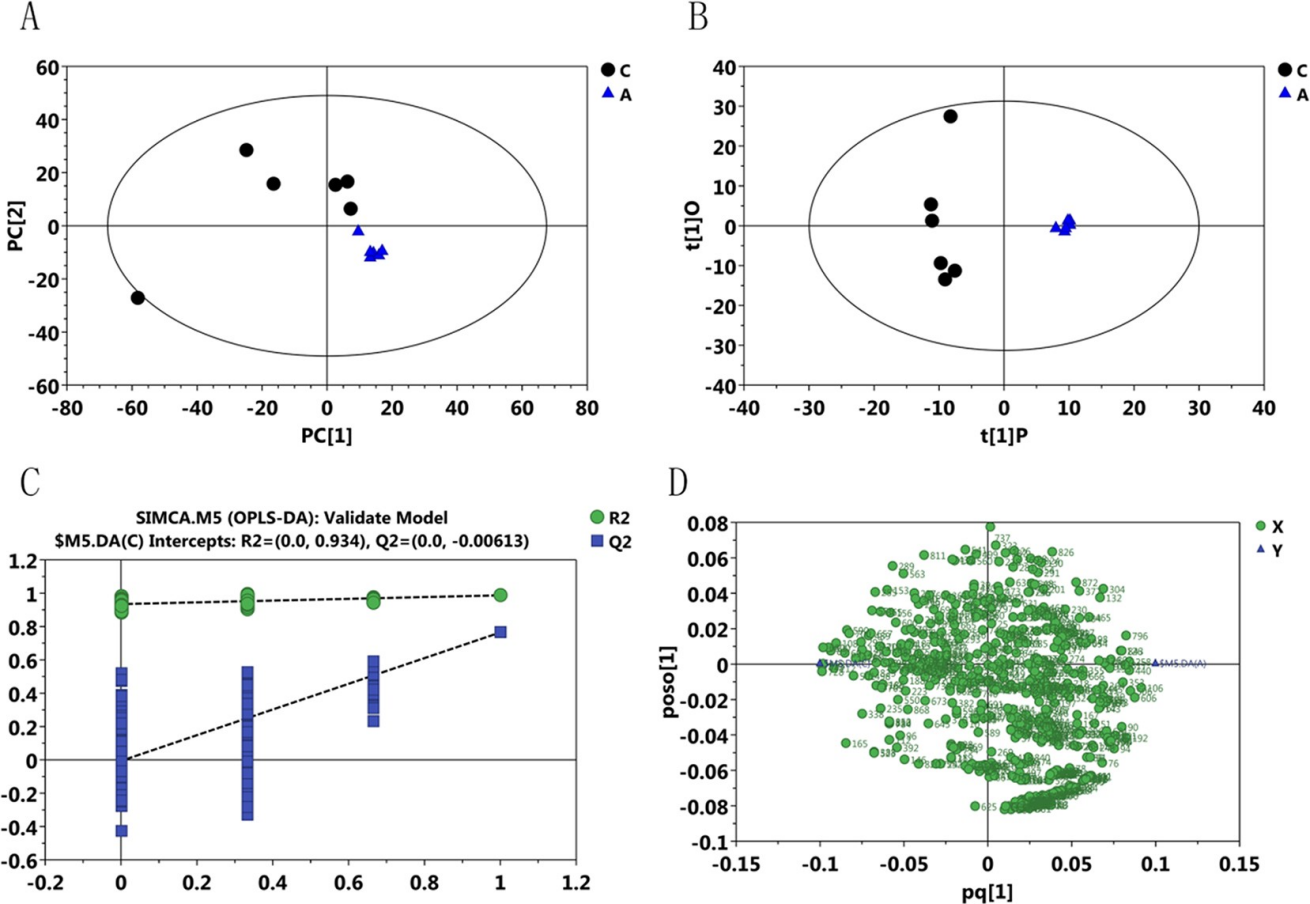
The multivariate data analysis among the C and A group (27°C vs 16°C). A, the scatter plot of the PCA model. B, The OPLS-DA model in order to identify the metabolites that differed significantly between the two groups. C, 200-permutation test was further applied to validate the OPLS-DA model, confirming the robustness of the model. D, loading plot based on the OPLS-DA model, and the metabolites located in the marginal area were potential discrepant metabolites.

**Fig 3:**
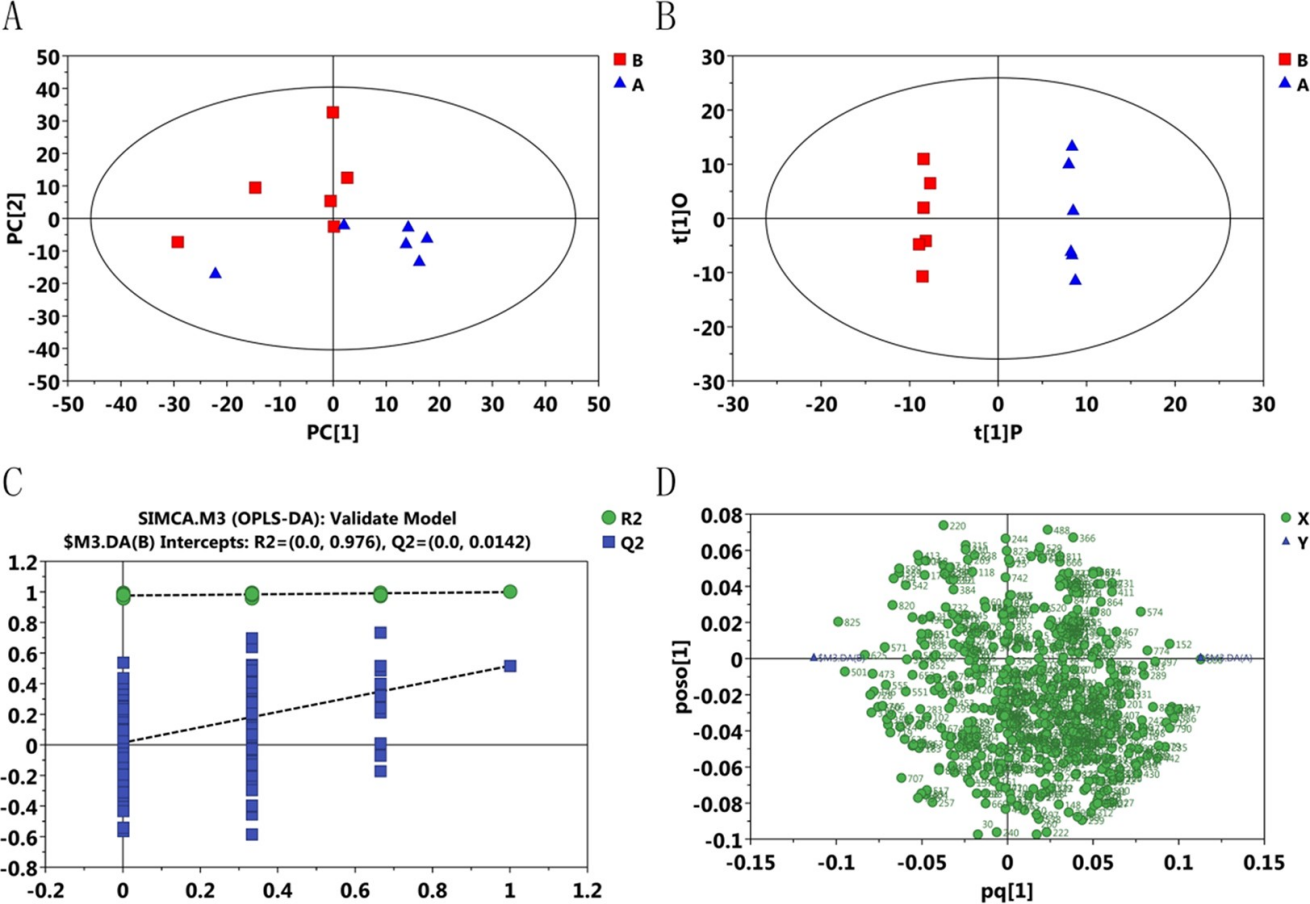
The multivariate data analysis among the B and A group (5°C vs 16°C). A, the scatter plot of the PCA model. B, The OPLS-DA model. C, 200-permutation test was further applied to validate the OPLS-DA model. D, loading plot based on the OPLS-DA model.

The differential metabolites between treatment and control groups were the key to explaining coping mechanisms or the occurrence of damage in black rockfish under thermal stress. To explore these differential metabolites, PCA models of the 16°C versus 27°C and 16°C versus 5°C groups were first applied (**Figs 2A** and **3A**, respectively). However, we did not obtain satisfactory results due to the high variation of six samples in one of the treatment groups. Similarly, OPLS-DA model was used to discriminate these two groups as shown in **Figs 2B** and **3B**. In the OPLS-DA model of 16°C versus 27°C, the parameters of the value of the first predicted component (R^2^Y = 0.987) and the value of the first orthogonal component (R^2^O = 0.367) were obtained and the value for the predictability of the model (Q^2^ = 0.765) was obtained through 7-fold cross validation, and in the OPLS-DA model of 16°C versus 5°C, values of R^2^Y = 0.999, R^2^O = 0.517, and Q^2^ = 0.517 were obtained. A test of 200 permutations was further applied to validate the model shown in **Figs 2C** and **3C**. The R^2^ and Q^2^ intercept values in the model of 16°C versus 27°C were 0.934 and 0.006, respectively, and in the model of 16°C versus 5°C were 0.976 and 0.014, respectively, and the low values of the Q^2^ intercept indicate the robustness of the models, and thus show a low risk of over fitting and are reliable. Based on the OPLS-DA model, a loading plot was constructed, which showed the contribution of variables to differences (**Figs 2D** and **3D)**. It also showed that the important variables were situated far from the origin, but the loading plot was complex because of many variables. To refine this analysis, the first principal component of variable importance projection (VIP) was obtained. The VIP values exceeding 1.0 were first selected as corresponding to differential metabolites.

**Fig 4:**
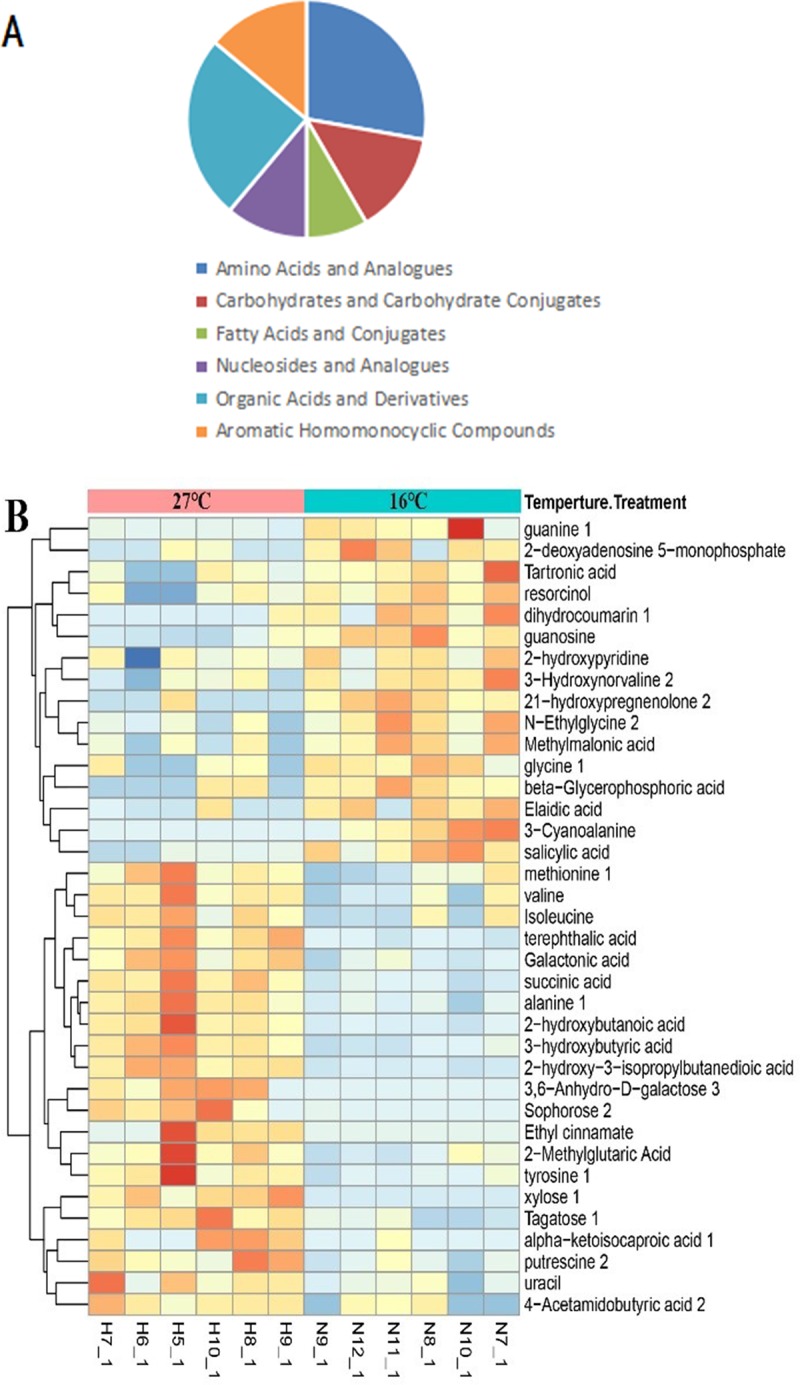
The profile of discrepant metabolites obtained from the OPLS-DA model of C vs A group (27°C vs 16°C) by the selected rule (VIP > 1). A, the category of the metabolites changed. B, the heat map representation of unsupervised hierarchical clustering applied in these metabolites.

**Fig 5:**
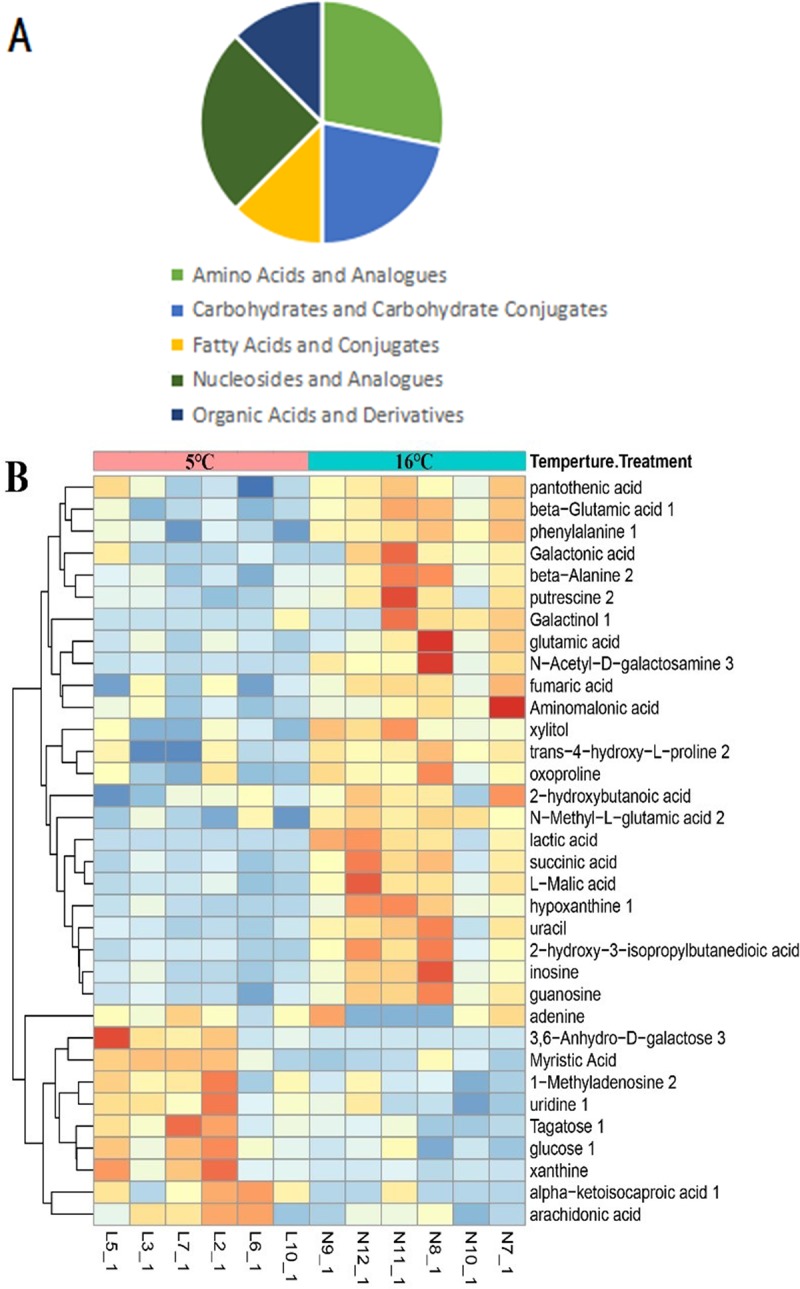
The profile of discrepant metabolites obtained from the OPLS-DA model of B VS A group (5°C vs 16°C) by the selected rule (VIP > 1). A, the category of the metabolites changed. B, the heat map representation of unsupervised hierarchical clustering applied in these metabolites.

Based on Student's T test, differential metabolites were chosen on the criteria that VIP > 1 and P < 0.05. The lists of discrepant metabolites in the 27 and 5°C groups compared to the 16°C group is shown in **S2** and **S3 Tables**, respectively. There were 36 annotated metabolites in all 92 discrepant peaks in response to 27°C treatment, and 34 annotated metabolites in all 59 discrepant peaks in response to 5°C treatment, and its categories are shown in **Figs 4A** and **5A**. Compared to the 16°C group, 21 metabolites were upregulated and 15 were downregulated in the 27°C group and 10 were upregulated while 24 were downregulated in the 16°C group. The annotated differential metabolite profiles in response to acute high and low temperature stress were displayed in heat maps (**Figs 4B** and **5B,** respectively). The number of the total changed metabolites due to the high temperature far exceeded those occurring due to the low temperature, and the number of upregulated metabolites showed a similar trend. The accumulation of metabolites therefore indicates a mismatch between different processes or a shift to other processes.

**Fig 6:**
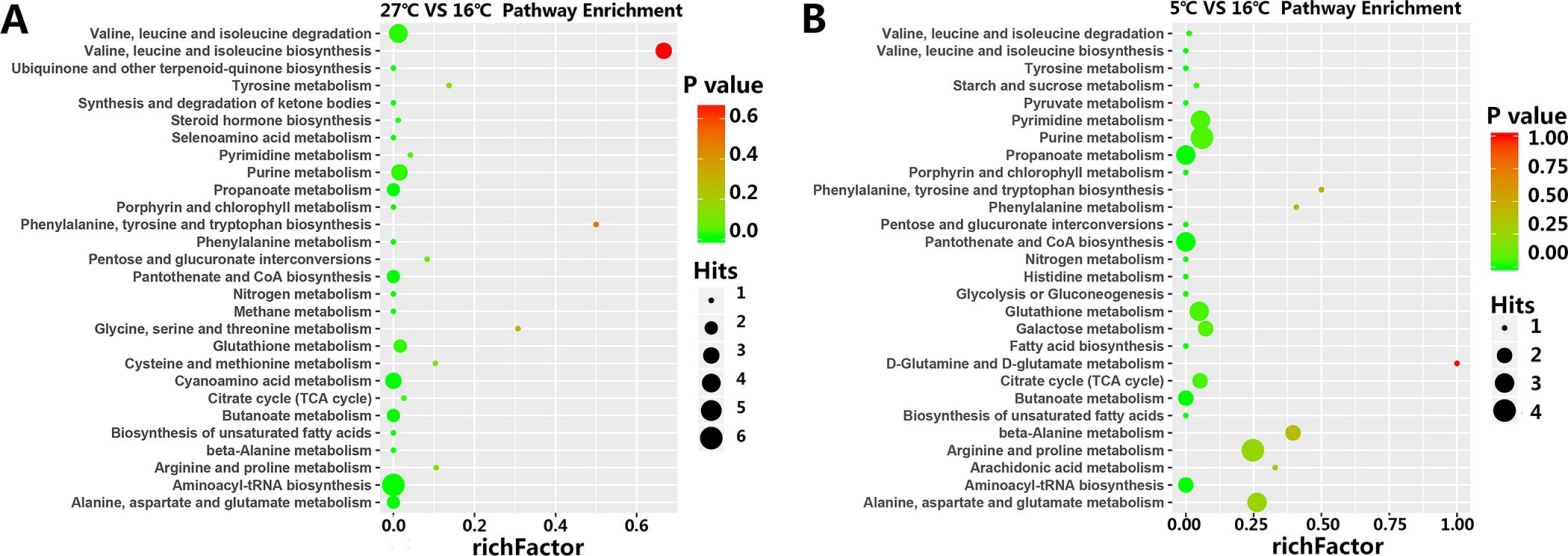
The pathway enrichment plot was based on the discrepant metabolites through the use of KEGG database. A, the pathway enrichment of black rockfish exposed to 27°C water compared to normal temperature of 16°C. B, the pathway enrichment of 5°C Group.

Furthermore, these differential metabolites were put into their respective biochemical pathways as described in the KEGG database, and enrichment factors of these pathways were calculated by MetaboAnalyst 3.0. The pathway enrichment results are shown in **Fig 6**. The analysis revealed that the metabolites that changed in response to high temperature participated in 28 target pathways (P < 0.05), including 13 pathways with an impact factor > 0. The first six highest impact pathways due to high temperature were all related to amino acid metabolism, including valine, leucine and isoleucine biosynthesis, phenylalanine, tyrosine and tryptophan biosynthesis, glycine, serine and threonine metabolism, tyrosine metabolism, arginine and proline metabolism, and cysteine and methionine metabolism. Compared to the 16°C group, the metabolites in the 5°C group had variation in 28 target pathways (P < 0.05), including 14 pathways whose impact factor was > 0, and the six biggest impact pathways were those related to D-glutamine and D-glutamate metabolism, phenylalanine, tyrosine and tryptophan biosynthesis, phenylalanine metabolism, β-alanine metabolism, arachidonic acid metabolism, alanine, aspartate and glutamate metabolism. The 27°C group and the 5°C group shared 19 similar pathways, which revealed that these pathways have a strong correlation to acute change in water temperature.

### The expression level of selected genes

#### *ub* gene expression analysis

**Fig 7:**
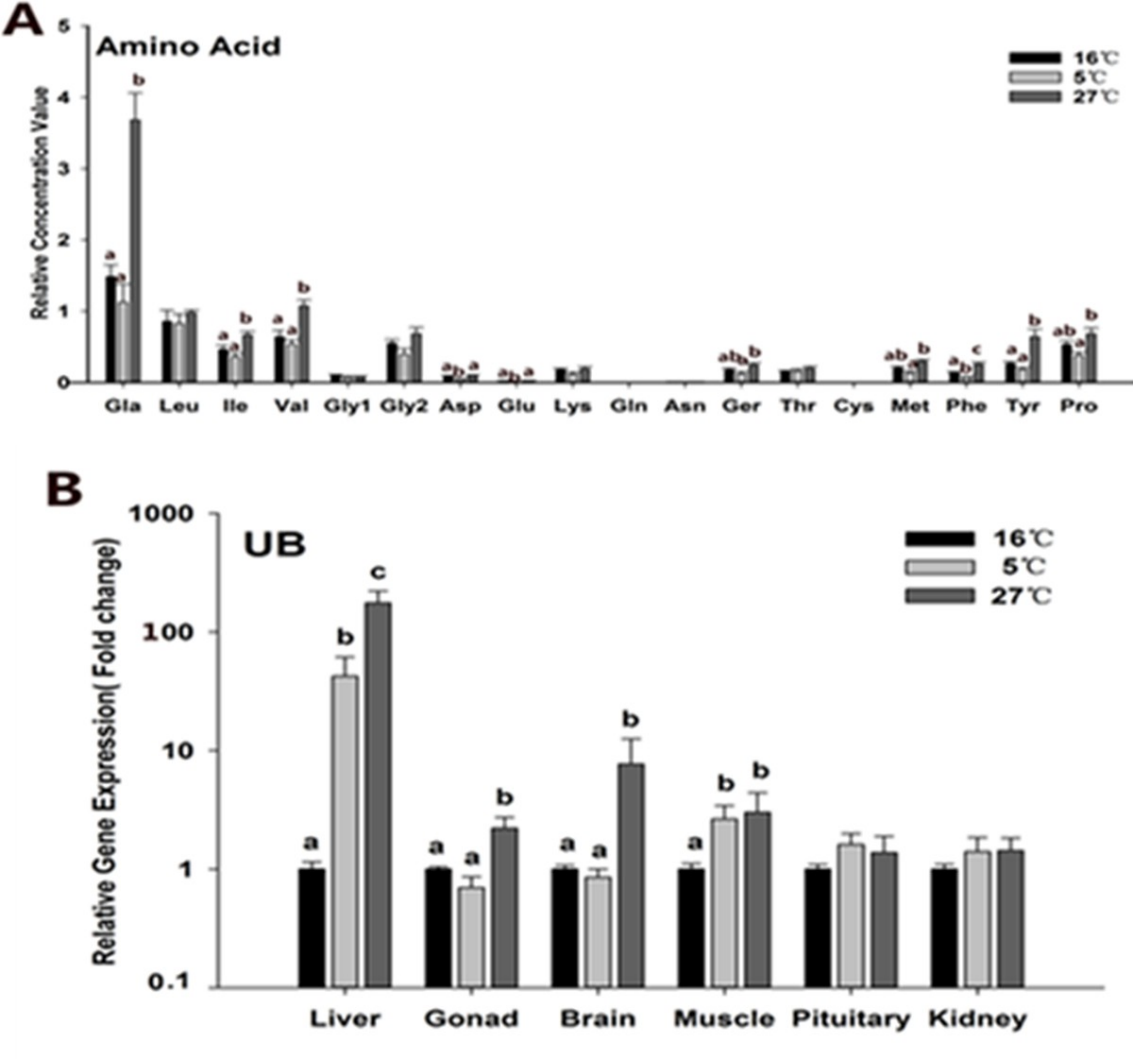
**The concentration of detected amino acids in serum (A) and expression levels of *ub* (B) in the above six tissues.** Values are given as mean ± SD, n = 6. Different letter on top of the column indicate significant differences (P < 0.05). No letter on column indicate no statistical change.

Because pathway enrichment mainly involved amino acid metabolism upon either high or low temperature stress, the detected amino acids were identified from the raw data and their relative concentrations among the three temperatures are shown in **Fig 7A.** The concentrations of alanine, isoleucine, valine, serine, methionine, phenylalanine, tyrosine and proline in the 27°C group had a significant increase compared to those in the 16 and 5°C groups, and the concentrations of amino acids in the 5°C group were not significantly different compared to those in the 16°C group except aspartic acid and glutamic acid, which were significantly decreased in the 27°C group (P < 0.05). From a global perspective, we hypothesized that upon high temperature stress, black rockfish increased the speed of protein degradation leading to increased concentrations of many amino acids in serum.

The *ub* expression was analyzed (**Fig 7B**). We showed that *ub* expression levels dramatically changed upon thermal stress in liver, gonad, brain, and muscle tissues. However, no statistically significant change was observed in pituitary and kidney tissues. Notably, liver *ub* gene expression in the 27°C group had an extremely significant increase in contrast to that in the 16°C group while expression in the 5°C group significantly decreased (P < 0.01). In gonad tissue, the expression level in the 27°C group was higher in comparison to that in the 16°C group (P < 0.05) while no significant change was detected in the 5°C group, with a similar trend for brain tissue. Overall, the above results of *ub* expression were consistent with the hypothesis that acute high thermal stimulation increased the speed of protein degradation.

#### *hif1α* and *hif2α* gene expression analysis

**Fig 8:**
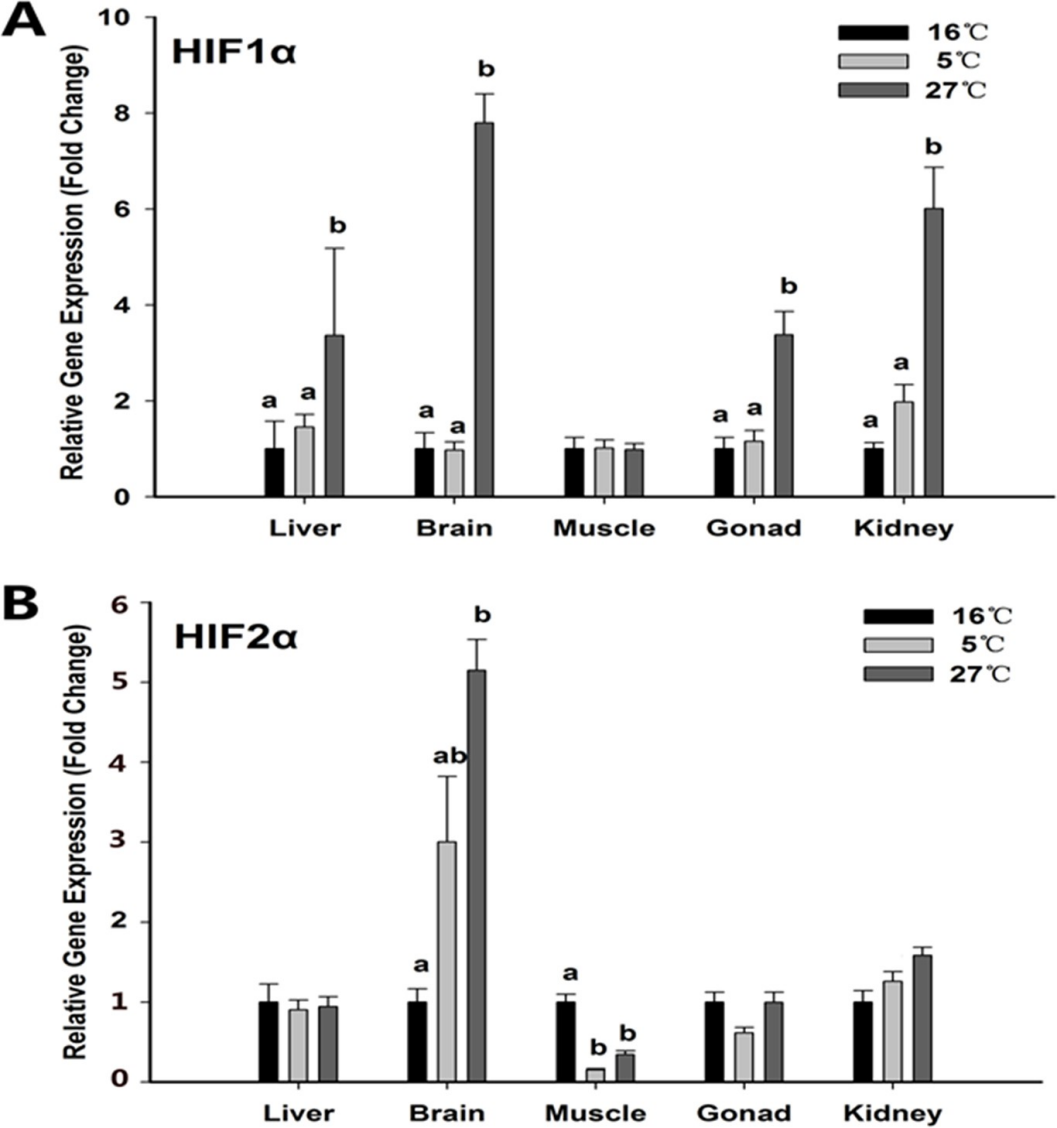
**Expression levels of *hif1* (A) and *hif2* (B) in the above five tissues.** Values are given as mean ± SD, n = 6. Different letter on top of the column indicate significant differences (P < 0.05). No letter on top of the column indicate no statistical change.

Metabolic rate is a measure of power utilization of an organism and is traditionally measured as the rate of oxygen consumption. Previous studies on thermal stress or hypoxia treatment suggest a similar physiological response to these stresses. Aquatic animals may act similarly. Therefore, we proposed that acute temperature change could affect the expression of HIFs and the expression results are shown in **Fig 8A** and **8B**. Compared to 16°C, high temperature treatment resulted in a statistically significant increase in the expression of *hif1α* in liver, brain, gonad and kidney tissues but not in muscle tissue (P < 0.05). However, no statistical change was observed in the 5°C group in all five tissues studied. Different from the *hif1α* expression pattern, the expression level of *hif2α* in muscle tissue of the 5°C group was decreased (P < 0.05) and no significant change was detected among the three groups in other tissues except the brain where *hif2α*expression increased in the 27°C group. The results indicated that acute thermal stimulation could affect the expression of *hif1α* and *hif2α* and the two genes had different expression patterns.

#### Analysis of *ldh* gene expression and some metabolites in relation to energy metabolism

**Fig 9:**
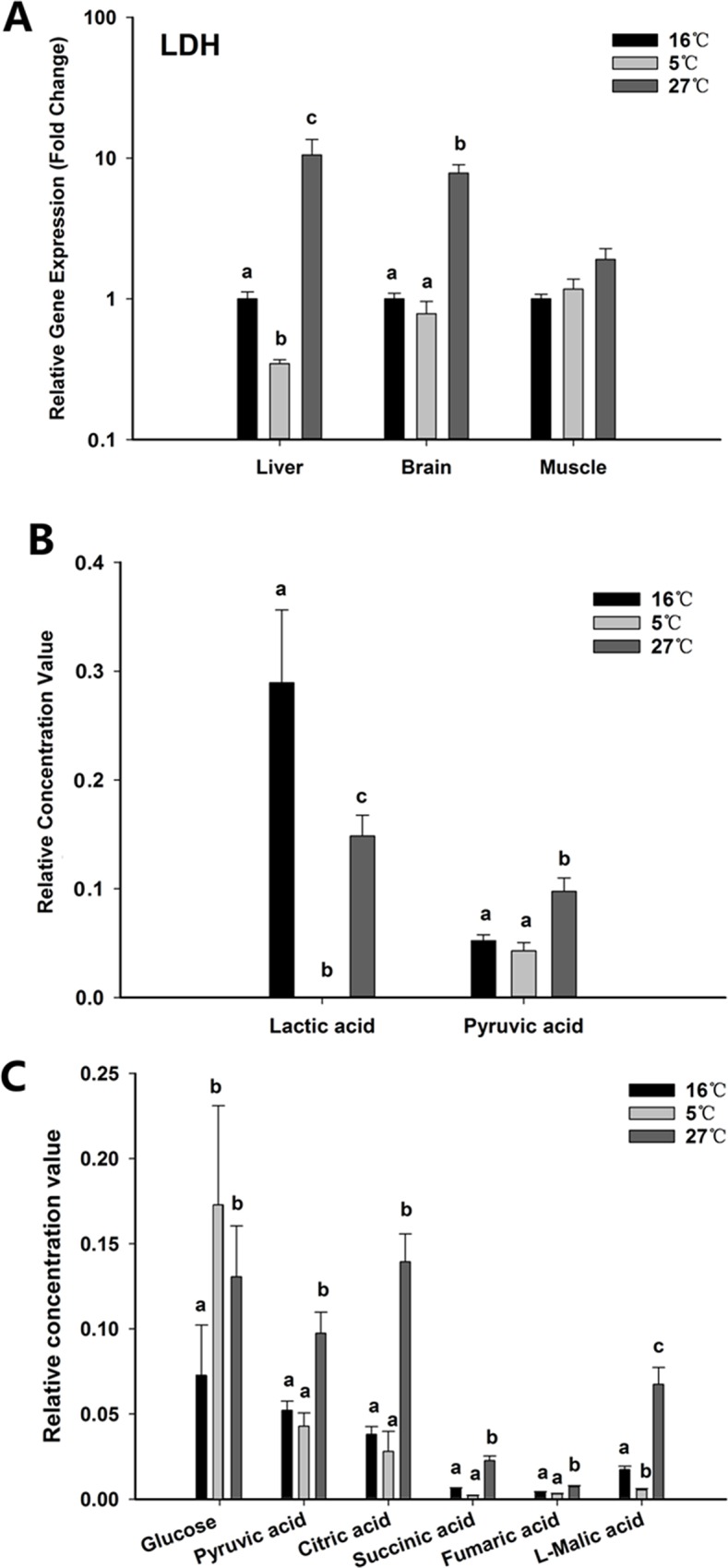
**Expression levels of ldh in the above three tissues (A), concentration of lactic acid and pyruvic acid (B), and concentration of glucose and some metabolites involved in TCA cycle in serum affected.** Values are given as mean ± SD, n = 6. Different letter on top of the column indicate significant difference (P < 0.05). No letter on column indicate no statistical change.

After the important discovery of HIFs, many researchers found that HIF1s regulate transcription of genes encoding glycolytic enzymes [30, 31] and LDH gene promoters contain essential binding sites for HIF1s [32]. Therefore, HIFs possibly act as a regulator of energy metabolism when the cells are stimulated by thermal or hypoxia stress or other stressors. **Fig 9A** shows *ldh* expression in response to different temperatures. The relative concentrations of pyruvate, lactate and other metabolites related to energy metabolism were calculated from the raw data of serum metabolomics and shown in **Fig 9B** and **9C**. In liver and brain, *ldh* expression was higher at 27°C than at 5 or 16°C. Compared to the 16°C group, lactic acid was significantly lower in both the 27 and 5°C groups (P < 0.05), even below the detection limit in the 5°C group. Pyruvate increased in the 27°C group. Additionally, glucose, citric acid, succinic acid, fumaric acid and L-malic acid in the 27°C group were significantly higher than those in the 16°C group. These metabolites were involved in the glycolytic pathway and citrate cycle (TCA cycle), leading to increased ATP production to overcome the adverse conditions.

#### *acac* gene expression analysis

**Fig 10:**
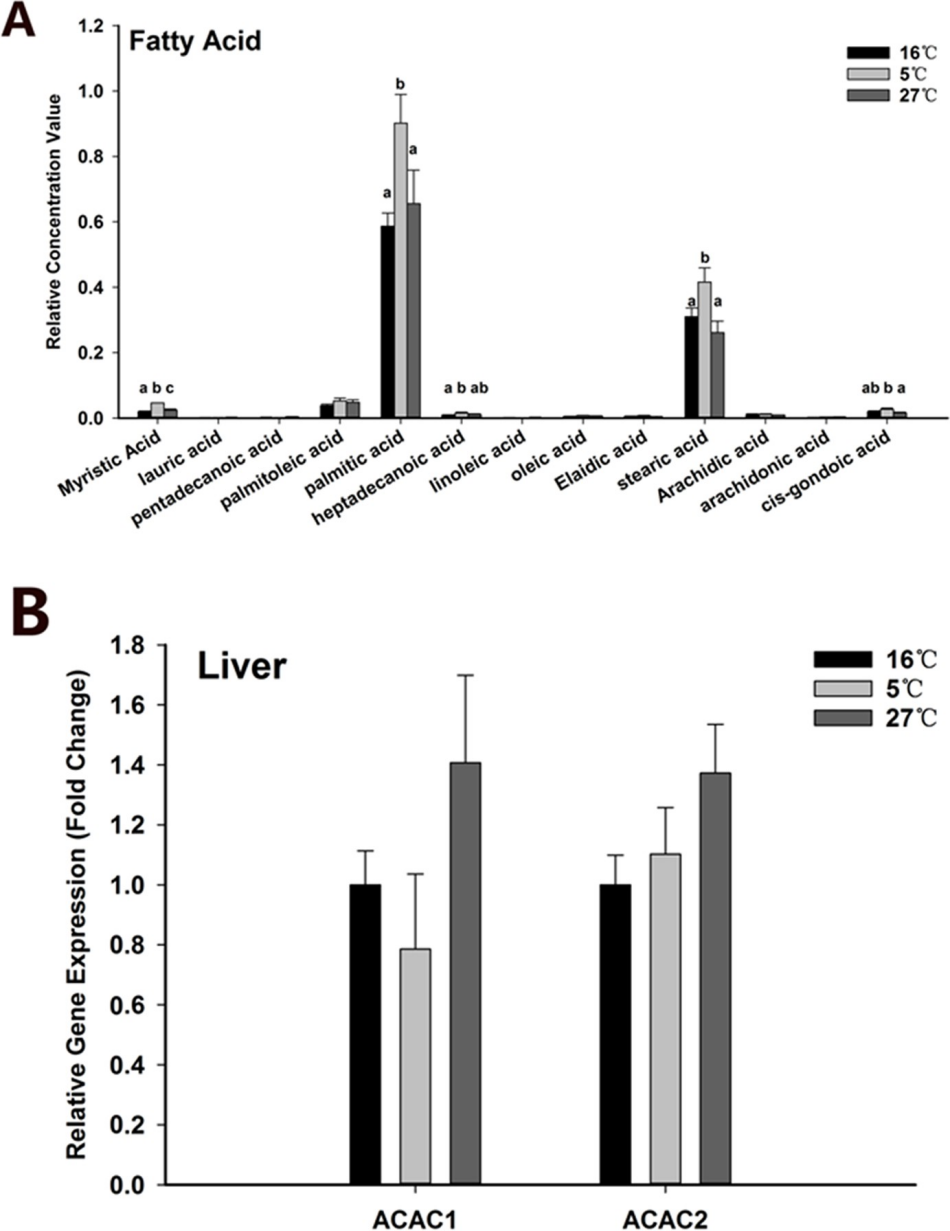
**The concentration of detected fatty acids in serum (A) and expression levels of *acac1* and *acac2* in liver (B).** Values are given as mean ± SD, n = 6. Different letter on top of the column indicate significant difference (P < 0.05). No letter on column indicate no statistical change.

Black rockfish is a cold resistant species; hence, it should be endowed with an intrinsic physiological system to adapt to the cold environment. In the pathway enrichment analysis of the 5°C group, fatty acid biosynthesis and arachidonic acid metabolism were enriched, indicating that some unsaturated fatty acids may play important roles in cold adaption. The relative concentrations of all long-chain fatty acids extracted from the raw data of serum metabolomics are shown in **Fig 10A.** In contrast to the 16°C group, levels of myristic, palmitic, stearic, and *cis*-gondoic acids were markedly increased in the 16°C group but not in the 27°C group. Palmitic acid is the first fatty acid produced during fatty acid synthesis and the precursor to longer chain fatty acids, and stearic acid is incorporated into some nonessential unsaturated fatty acids; hence, these two fatty acids are crucially involved in lipid metabolism. We hypothesized that acute cold exposure induced *acac* expression to enhance fatty acid synthesis. Liver is the main tissue for fatty acid synthesis. Therefore, the expression levels of two members of the ACAC gene family in liver were analyzed and the data are shown in **Fig 10B**. Unexpectedly, we observed no significant effects from thermal stress.

## Discussion

We used GC-TOF-MS as our main tool for metabolite identification, which has the advantages of high resolution and sensitivity, detecting more metabolites, and easy quantification and qualitative analysis with professional NIST databases, although sample preparation can be complex and lengthy. In this study, we demonstrated the utility of a GC-TOF-MS-based metabolomics method to assess changes in the metabolome of black rockfish. We demonstrated differential metabolite changes in serum in response to acute temperature shifts. Methods with different mathematic principles were used to assess statistical significance, with the OPLS-DA specializing in high correlative multivariate analysis and traditional one-way ANOVA being used for the analysis of independent individual variables. Therefore, in our study, some important metabolites to be discussed and refined from the raw metabolomics data identified by one-way ANOVA were not found in the library of differential metabolites obtained from the OPLS-DA model. T.V. Bagnyukova showed that a short-term exposure to warm temperature disturbed several oxidative stress markers within 1–120 h[33]. Yin & Blaxter and Bøhle observed 50% mortality after 24 h of exposure[34, 35]. Chavin & Young (1970) reported that significant effects on plasma glucose levels were not apparent in *Carassius auratus* until 15 min after temperature change [36]. Overall, on the basis of serum metabolomics combined with key gene expression analysis, our study could explain the overall physiological effects of acute thermal change in black rockfish.

### Energy metabolism

At a critical temperature (Tc) the exacerbated mismatch between oxygen supply and demand finally leads to the onset of anaerobic metabolism[37]. Glucose was found to be increased after acute low and high thermal stress. In the warmth, free amino acids can also fuel glucose by means of glycogenolysis[38] to support metabolism under increased energy demands and, more likely, functional hypoxia. Windisch et al. showed that respiratory chain capacities increased accompanied by a shift from lipid to carbohydrate catabolism in the liver of the Antarctic eelpout *Pachycara brachycephalum* after long-term acclimation to 5°C[39]. This increase was expected since the adverse environment forced black rockfish to switch to gluconeogenesis or glycogenolysis, resulting in an accumulation of glucose in serum that was utilized by somatic cells. In contrast, both high and low temperature treatments induced a decrease in serum lactate concentration. Although the predominant fuels for energy production *in vivo* are long chain fatty acids and glucose, lactate is an intermediate of glucose/amino acid metabolism [40]. Decreased lactate might be due to usage of lactate by cardiac tissue for producing energy when energy demand is high, as with acute thermal stress. We also found that citric, succinic, fumaric and malic acids, which are all involved in the TCA cycle, were increased in serum in the 27°C group, but there was no change in the 5°C group. The TCA cycle provides the reduction equivalents for terminal oxidation during respiratory change; increased TCA cycle activity indicated an elevated energy demand and higher metabolic rate.

Marshall, D. J. et al. proposed a new universal temperature dependence (UTD) model, which explains the complexity of metabolism in relation to temperature range experienced and the degree of metabolic depression[41]. It is proposed that the metabolic rate of organisms is driven directly by the kinetic energy of the cell; a higher temperature leads automatically to a higher metabolic rate in all organisms [42]. Though the theory that metabolic rate is driven mechanistically by temperature is incompatible with what we know of cellular physiology and the molecular mechanisms of evolutionary adaptation to temperature, the metabolic rate in ectotherms is widely observed to covary with environmental temperature [43]. Like most biochemical reactions, the reactions of aerobic metabolism were directly influenced by environmental temperature [42], so organisms must have mechanisms to cope with these effects to preserve energy generation across temperatures [44]. Thus, a high metabolic rate requires large amounts of energy to maintain, which leads to an increasing demand for oxygen and, subsequently, a decreased cellular oxygen partial pressure [45, 46].

In the present study, HIF1*α* was found to be significantly increased by acute high temperature changes in brain, liver, gonad and kidney tissues. HIF1 is a transcription factor regulating cellular response to hypoxia stress, first found to accelerate erythropoietin generation. It also has an important function in improving the gene expression of glucose transporters and glycolytic enzymes to cover oxygen shortages, since the promoters of glucose transporters and glycolytic enzyme genes contain essential binding sites for HIF1 [47–49]. There are many studies about HIF-1α mRNA levels in fishes exposed to acute and chronic hypoxia [50, 51]. We also found that high thermal stress induced *ldh* expression in liver and brain tissue, and *ldh* is upregulated by HIF1, which causes the conversion of lactate to pyruvic acid for the TCA cycle. Although the effects of temperature on HIF1 function have not been studied thoroughly in poikilothermic animals, Rissanen found that in crucian carp (*Carassius carassius*), temperature caused increases in HIF1*α* protein levels and especially the DNA-binding activity of HIF1 [52]. This can nicely explain our proposed hypothesis that acute high temperature change and hypoxia stress involve the same physiological acclimation, which involves identical high energy and oxygen demands and induction of mass expression of HIF1. Generally, fish were more active in hypoxia than when the same fish were startled in normoxia, which positively correlated with metabolic rate[53]. However, only brain tissue was observed to have an increasing trend in the expression of HIF2α in response to acute high thermal stress, which indicated that different types of HIF-αs have different functions.

In contrast, when the black rockfish was quickly placed in cold water, the above metabolites involved in the TCA cycle did not change compared to those of the control group. There was a marked decrease in the concentration of malic acid which plays an important role in the TCA cycle and the malate-aspartate shuttle used to transfer NADH to the inner mitochondrial membrane. We also found no increase in the expression level of *hif1α* and *ldh*; all of this indicated that acute cold treatment did not cause an increasing demand for ATP, as in mammals, to maintain normal body temperature. Generally, as for ectotherms, there are two strategies of response to cold acclimation through changes in metabolism: (1) an increase in metabolism to compensate for temperature-mediated decreases in the metabolic rate, or (2) suppression of the metabolic rate to reduce energy consumption [54].

Cold-tolerant fish usually maintain reduced metabolic rates when exposed to cold water. Adaptations for coping with hypoxia and anoxia among overwintering freshwater fishes may include metabolic depression, a decrease in blood O_2_ affinity, microhabitat selection, air breathing, short-distance migration, biochemical modifications aimed at adjusting glycolytic rates, and alcoholic fermentation[55]. The cunner (*Tautogolabrus adspersus*) decreases its metabolic rate during seasonal exposure to winter temperatures, with routine oxygen consumption decreased to Q_10_ of 10.4 when acclimated to cold temperature [56]. In Atlantic killifish (*Fundulus heteroclitus*), routine oxygen consumption rapidly decreases in white muscle during exposure to low temperature [57]. It is clear that in fully acclimated individuals, standard and active metabolic rates decrease with Q_10_ of 1.6–2.0 as water temperature is reduced [58]. Thus, black rockfish may utilize the strategy of suppressing their metabolic rate when exposed to low water temperature, contributing to survival during winter.

### Amino acid metabolism

From the pathway enrichment map, interestingly, we found that the six largest impact pathways in the 27°C group and the four largest impact pathways in the 5°C group were all related to amino acid metabolism. Eight amino acids significantly increased in the 27°C group and four amino acids decreased in the 5°C Group, indicating that amino acids changed in different ways upon heat or cold stress.

One reason for the increase of free amino acids in the 27°C group may be protein degradation. Thus, we examined the expression level of *ub*. We fund that the expression of *ub* was increased in liver, gonad, and brain tissues when black rockfish were quickly exposed to high temperature water. Artigauel et al. identified a number of genes related to ubiquitination and protein degradation that were upregulated when king scallop (*Pecten maximus*) are exposed to long-term heat stress [59]. Feidantsis et al. found that increasing ubiquitinated protein levels occur in the heart and liver during combined warming and hypercapnia in the gilthead seabream (*Sparus aurata*) [60]. Madeira et al. also found the increase in total ubiquitin at higher temperatures in *Sparus aurata*, which indicated protein damage [61]. Lipids are major targets during heat-driven oxidative stress; polyunsaturated fatty acids are degraded to a variety of products, such as aldehydes and can damage proteins [62]. Undesirable accumulation of ROS can cause oxidative damage to cell components including proteins, lipids, and nucleic acids, and high amounts of ROS usually lead to increased levels of carbonylated proteins[62, 63]. Possibly, higher turn-over due to protein damage from oxidative stress and ROS in some tissues caused by high temperature may account for the increase of basic amino acids in serum.

However, low temperature did not cause an increase in serum amino acid levels, even with a decrease in aspartic acid and glutamic acid. Likewise, we found no changes in *ub* expression level in the six tissues studied except for a decrease *ub* expression in muscle tissue. These data indicated that acute cold exposure did not perturb amino acid metabolism. Nevertheless, some studies demonstrated that both heat stress and low temperature can induce protein denaturation [64, 65]. Todgham et al. found higher levels of damaged proteins in Antarctic fishes when compared to fish from more temperate environments through measuring levels of Ub-conjugated proteins [66].

From the perspective of individual amino acids, the striking result was that high temperature induced an extremely significant increase of alanine, but low temperature did not. Alanine accumulation is now universally accepted as the first signal of stress in a variety of organisms [67]. Alanine has been found to modulate the peripheral nervous system [68] through activation of glycine receptors. Glycine is an inhibitory neurotransmitter, helping organisms to overcome anxiety and coping with the effects of stress [69]. It has also been speculated that alanine can stimulate the genes encoding the synthesis of stress proteins. However, there was no alanine accumulation induced by cold stress, which could be attributed to different temperature stress responses. Increasing concentrations of alanine in serum could be generated from protein degradation and other amino acids through alanine transaminase. The Cahill cycle, also known as the alanine cycle or glucose-alanine cycle, is the series of reactions in which amino groups and carbons from tissues are transported to the liver. We suggest that the serum alanine surge caused by heat stress was involved in the Cahill cycle that shuttled the alanine generated in tissues to the liver to synthesize pyruvate to meet the increasing energy demand.

### Fatty acid metabolism

Because of the high cold tolerance of black rockfish, there were few physiological variations affected by acute cold stress, compared to heat stress, as illustrated from the PCA score plot (**Fig 1B**). From the results of the serum metabolome, we found that the pathway of fatty acid biosynthesis and arachidonic acid metabolism were enriched in the 5°C group. Myristic acid, palmitic acid, stearic acid, and *cis*-gondoic acid in serum were increased in response to low temperature, likely not due to enhanced fatty acid biosynthesis since *acac* expression was not changed. Increased lipolysis might be a contributing factor. Apart from carbohydrates, lipid oxidation accounts for an important fraction of energy metabolism in most tissues [70]. Driedzic et al. reported that an increase in 3-hydroxyacyl CoA dehydrogenase activity correlates with an increase in cardiac output in a cold environment, indicating enhanced oxidation of fatty acids [71]. Similarly, Kyprianou et al. showed increased activities of 3-hydroxyacyl CoA dehydrogenase in heart and red muscle and a transient increase in the levels of triglycerides in the plasma of gilthead seam transferred from an 18°C to a 10°C tank [72]. In response to cold stress, polar and nonpolar lipid storage changes, (for example, accumulation of glycerol), glycogen was accumulated, and increased protein levels may be related to the energy metabolism requirements during the period of the cold stress, which could be less energy intensive [73–76]. The capacity for lipid oxidation and storage has been found to have an increase during laboratory acclimation and seasonal cold acclimatization in rainbow trout [77, 78]. In many species of north temperate teleosts, seasonal decreases in temperature in summer or in winter induce an enhancement of aerobic-based fatty acid oxidation in the heart [79, 80]. It is well known that the ability of lipid utilization in fish is higher than in mammals. The storage of fat may allow fish to survive over a cold winter through the process of β-oxidation. Hence, the black rockfish may strengthen fatty acid oxidation to supply energy in response to both acute cold stress and chronic cold exposure.

It has been suggested that decreased temperature results in decreased membrane fluidity. To maintain proper membrane fluidity, fish generally enhance long chain polyunsaturated fatty acids levels, which has been called ‘homeoviscous adaptation’ [43]. In several fishes, including common carp [81], rainbow trout [82], gilthead sea bream [83], and milkfish [84], increased levels of unsaturated fatty acids in response to cold stress are observed. The levels of unsaturated fatty acids in an organism depend not only on dietary intake but also on desaturation of fatty acids. Hsieh et al. found that changes in monounsaturated fatty acids are highly correlated with those of stearoyl-CoA desaturase activities in hepatic microsomes of milkfish during cold acclimation. In the present study, only serum *cis*-gondoic acid levels were increased in unsaturated fatty acids in response to acute cold exposure. Therefore, in black rockfish, further studies are needed to determine whether levels of long chain polyunsaturated fatty acids were increased under exposure to cold environments for different lengths of time.

## Conclusions

Although there are several reports on metabolic and molecular responses to thermal stress in several fishes, our knowledge of black rockfish responses to thermal stress is very limited. In this study, we focused on the metabolic and molecular responses of black rockfish subjected to thermal stress. From serum metabolome data, we hypothesized that expression of several genes was affected by thermal stress. Significant changes in the metabolites associated with energy, amino acid, and fatty acid metabolism were observed, as were changes in expression levels of several genes related to specific metabolic pathways. We showed that the use of GC-TOF-MS together with advanced data analysis is useful for assessing the overall metabolic effect of thermal stress in black rockfish. A deficiency of this study is that we only studied a single time point. Further studies on short-term thermal stress are warranted.

## Supporting information

S1 Table The sequence of gene primers which were used in quantitative RT-PCR. (PDF)

S2 Table The list of discrepant metabolites from the result of OPLS-DA model in A VS C pattern (A, 16°C, and B, 27°C), and the metabolites which was marked red color is down-regulated compared to control group and correspondingly blue color is up-regulated. (PDF)

S3 Table The list of discrepant metabolites from the result of OPLS-DA model in A VS B pattern (A, 16°C, and B, 5°C), and the metabolites which was marked red color is down-regulated compared to control group and correspondingly blue color is up-regulated. (PDF)
