# Supplementary material for: The impact of acute thermal stress on the metabolome of the black rockfish (Sebastes schlegelii)
Source: PLoS One. 2019 May 24;14(5):e0217133. doi: 10.1371/journal.pone.0217133 (PMC6534312; doi:10.1371/journal.pone.0217133)
Supplement: S1 Table — (PDF) [file pone.0217133.s001.pdf]

Genes	Primers (5’-3’)
UB	F: GAGCCAAGTGACACCATT
	R: GGATGTTGTAGTCGGAGAG
HIF1α	F:ATTGTCACTCCCTATCCAAG
	R:CAAAACCCAGATGCTGCTAT
HIF2α	F:CGATGAAAACGGACAGATGG
	R:TGTTGTGCTCGGGACAGGAAT
LDH	F: GAAAATCCCAAACTGCCTGA
	R:TCCATCACATCAACCAGGGC
ACAC1	F: AAATCGTCAGTGTTCCGACTG
	R: TTCACCATGACGGGGTAGC
ACAC2	F: GTCAGCACTCTGAGCCACAA
	R: GAAAATCCCAAACTGCCTGA
18S	F: CCTGAGAAACGGCTACCATC
	R: CCAATTACAGGGCCTCGAAAG
