# Supplementary material for: The impact of acute thermal stress on the metabolome of the black rockfish (Sebastes schlegelii)
Source: PLoS One. 2019 May 24;14(5):e0217133. doi: 10.1371/journal.pone.0217133 (PMC6534312; doi:10.1371/journal.pone.0217133)
Supplement: S2 Table — (PDF) [file pone.0217133.s002.pdf]

Peak	Similarity	MEAN A	MEAN C	VIP	P-VALUE
methionine 1	944	0.20315	0.29787	1.53849	0.02914
Isoleucine	944	0.46318	0.66679	1.53216	0.02466
valine	934	0.64085	1.07492	1.79986	0.00534
tyrosine 1	929	0.26475	0.64277	2.02595	0.01213
uracil	927	0.02041	0.04052	1.53415	0.01354
succinic acid	926	0.00541	0.02261	2.19822	0.00111
guanosine	925	0.08756	0.02641	1.97157	0.00073
2-hydroxybutanoic acid	899	0.00627	0.03012	2.25343	0.00317
glycine 1	874	0.09784	0.03972	1.48586	0.02474
alanine 1	870	1.48429	3.68371	2.05706	0.00032
Elaidic acid	865	0.00453	0.00090	1.71199	0.01685
putrescine 2	859	0.01528	0.03744	1.70811	0.00836
Galactonic acid	840	0.00046	0.00185	1.13359	0.00118
xylose 1	759	0.00000	0.00138	2.36347	0.00148
3-hydroxybutyric acid	742	0.12908	0.43673	2.16853	0.00062
alpha-ketoisocaproic acid 1	736	0.00012	0.00111	1.19278	0.04086
3-Hydroxypyridine	736	0.02287	0.01575	1.47796	0.04536
beta-Glycerophosphoric acid	722	0.00196	0.00066	1.67460	0.01978
conduritol b epoxide 2	658	0.00463	0.05571	1.45098	0.00638
Tagatose 1	614	0.00170	0.00829	1.36816	0.00057
3,6-Anhydro-D-galactose 3	570	0.00000	0.00337	1.92599	0.01493
Sophorose 2	549	0.00005	0.00351	1.63457	0.01593
terephthalic acid	543	0.00016	0.00260	1.59254	0.00207
guanine 1	510	0.00160	0.00020	1.36639	0.03432
2'-deoxyadenosine 5'-monophosphate	480	0.00205	0.00041	1.18536	0.01498
21-hydroxypregnenolone 2	468	0.00249	0.00045	1.92160	0.00396
3-Cyanoalanine	466	0.00084	0.00000	2.02088	0.01919
3-Hydroxynorvaline 2	463	0.01922	0.01141	1.51345	0.01504
2-hydroxy-3-isopropylbutanedioic acid	448	0.01213	0.13490	2.27662	0.00022
Neohesperidin	429	0.00117	0.00041	1.76271	0.02959
Methylmalonic acid	413	0.01030	0.00812	1.59938	0.01665
N-Ethylglycine 2	377	0.01620	0.01307	1.75539	0.00753
resorcinol	362	0.00412	0.00203	1.15303	0.02334

Peak	Similarity	MEAN A	MEAN C	VIP	P-VALUE
DL-Anabasine 1	355	0.00117	0.00044	1.22364	0.00181
2-Methylglutaric Acid	338	0.02306	0.03925	1.81114	0.01492
4-Acetamidobutyric acid 2	320	0.00161	0.00381	1.39388	0.02175
dihydrocoumarin 1	284	0.00229	0.00031	1.59274	0.01895
Ethyl cinnamate	268	0.00000	0.00045	1.79374	0.03945
Tartronic acid	266	0.00053	0.00023	1.18837	0.03380
D-erythronolactone 2	243	0.00947	0.00371	1.18174	0.00910
3-aminopropionitrile 1	233	0.00022	0.00052	1.17313	0.03013
salicylic acid	228	0.00136	0.00080	1.93098	0.00136
