# Supplementary material for: The impact of acute thermal stress on the metabolome of the black rockfish (Sebastes schlegelii)
Source: PLoS One. 2019 May 24;14(5):e0217133. doi: 10.1371/journal.pone.0217133 (PMC6534312; doi:10.1371/journal.pone.0217133)
Supplement: S3 Table — (PDF) [file pone.0217133.s003.pdf]

Peak	Similarity	Mean A	Mean B	VIP	P-Value
xylitol	954	0.02198	0.01394	1.90605	0.00970
inosine	932	0.11484	0.04574	2.28732	0.00921
phenylalanine 1	928	0.14302	0.07315	2.10955	0.00047
uracil	927	0.02041	0.00583	2.21973	0.00648
oxoproline	927	1.20998	0.78486	1.74168	0.02793
succinic acid	926	0.00541	0.00207	2.23490	0.00614
guanosine	925	0.08756	0.02969	2.04349	0.00072
Myristic Acid	911	0.02251	0.03866	1.82087	0.01696
glucose 1	909	0.07253	0.17280	1.73169	0.01575
2-hydroxybutanoic acid	899	0.00627	0.00438	1.50948	0.04209
beta-Alanine 2	898	0.00709	0.00310	1.99059	0.00695
fumaric acid	879	0.00420	0.00307	1.89923	0.01062
Aminomalonic acid	879	0.07197	0.03897	1.86430	0.02908
L-Malic acid	878	0.01582	0.00540	2.30977	0.00417
putrescine 2	859	0.01528	0.00556	1.90437	0.01441
Galactonic acid	840	0.00046	0.00011	1.46971	0.03791
trans-4-hydroxy-L-proline 2	840	0.10020	0.04389	1.30007	0.01106
glutamic acid	832	0.01986	0.00898	1.83104	0.04379
arachidonic acid	831	0.00209	0.00322	1.50544	0.04488
xanthine	749	0.00121	0.00820	2.01387	0.03183
N-Methyl-L-glutamic acid 2	747	0.00418	0.00200	2.07519	0.00117
alpha-ketoisocaproic acid 1	736	0.00012	0.00067	1.88626	0.01857
hypoxanthine 1	736	0.06663	0.02009	2.34926	0.00809
pantothenic acid	697	0.00271	0.00146	1.01936	0.03104
lactic acid	642	0.24109	0.00000	2.31961	0.00736
Tagatose 1	614	0.00170	0.00673	1.42845	0.01643
N-Acetyl-D-galactosamine 3	610	0.00568	0.00235	2.40146	0.00947
uridine 1	588	0.00476	0.00799	1.76500	0.02795
3,6-Anhydro-D-galactose 3	570	0.00000	0.00110	2.28219	0.02664
Galactinol 1	534	0.00283	0.00043	1.44521	0.04458
beta-Glutamic acid 1	482	0.00919	0.00224	1.45300	0.00077
2-hydroxy-3-isopropylbutanedioic acid	448	0.01213	0.00250	2.27954	0.00833
adenine	490	0.00147	0.00199	1.51888	0.51175
Neohesperidin	429	0.00117	0.00028	1.97591	0.00364
1-Methyladenosine 2	245	0.00378	0.00686	1.64064	0.02675
